# Accelerated First‐Principles Exploration of Structure and Reactivity in Graphene Oxide

**DOI:** 10.1002/anie.202410088

**Published:** 2024-11-13

**Authors:** Zakariya El‐Machachi, Damyan Frantzov, A. Nijamudheen, Tigany Zarrouk, Miguel A. Caro, Volker L. Deringer

**Affiliations:** ^1^ Inorganic Chemistry Laboratory Department of Chemistry University of Oxford Oxford OX1 3QR United Kingdom; ^2^ Department of Chemistry and Materials Science Aalto University 02150 Espoo Finland

**Keywords:** carbon materials, computational chemistry, graphene, machine learning, neural-network potentials

## Abstract

Graphene oxide (GO) materials are widely studied, and yet their atomic‐scale structures remain to be fully understood. Here we show that the chemical and configurational space of GO can be rapidly explored by advanced machine‐learning methods, combining on‐the‐fly acceleration for first‐principles molecular dynamics with message‐passing neural‐network potentials. The first step allows for the rapid sampling of chemical structures with very little prior knowledge required; the second step affords state‐of‐the‐art accuracy and predictive power. We apply the method to the thermal reduction of GO, which we describe in a realistic (ten‐nanometre scale) structural model. Our simulations are consistent with recent experimental findings, including X‐ray photoelectron spectroscopy (XPS), and help to rationalise them in atomistic and mechanistic detail. More generally, our work provides a platform for routine, accurate, and predictive simulations of diverse carbonaceous materials.

## Graphical abstract

Emerging machine‐learning (ML) approaches to computational chemistry provide atomistic insight into graphene oxide. This work showcases how ML potentials can be fitted by combining accelerated first‐principles molecular dynamics and graph neural‐network models. Large‐scale simulations, driven by the resulting potential, reveal how functional groups in a graphene oxide sheet evolve and graphitic domains form during thermal reduction.

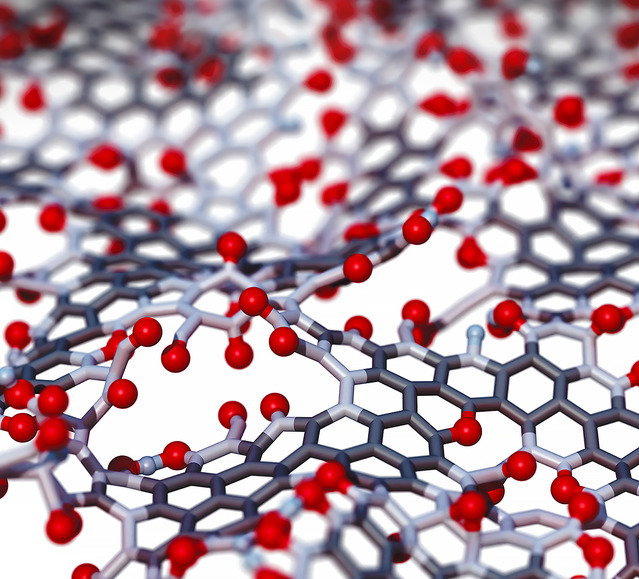

Graphene oxide (GO) is a summary term for a range of layered materials created by reacting graphite with aggressive agents, such as KMnO_4_, typically followed by partial reduction and sometimes functionalisation.[^1^, ^2^, ^3^, ^4^] Today, GO materials can be controllably prepared^[5]^ and find emerging applications in catalysis,^[6]^ membranes,^[7]^ electronics,^[8]^ and photonics.^[9]^ Despite decades of work, however, the precise chemical structure of these materials has remained elusive. The ordered regions of GO sheets can be directly visualised using high‐resolution electron microscopy,[^10^, ^11^] but the nature of the more disordered regions can only be inferred from indirect observations, such as vibrational and NMR spectroscopy. The properties of GO materials cannot be unambiguously linked to chemical structure if this structure itself is not precisely known (which functional groups are present; in what amounts?).

To complement experimental techniques, GO has been widely studied by computational chemistry methods. For example, Kumar et al. combined reactive‐force‐field simulations with density‐functional theory (DFT) to show how varying functional groups affect the stability and electronic structure of thermally reduced graphene oxide (rGO), and how rGO forms graphitic and oxidised domains during thermal annealing.[^16^, ^17^] Atomistic modelling of rGO further revealed that defects formed during thermal reduction can lead to pores for applications in water desalination and natural gas purification.^[18]^ Explicit water molecules have been incorporated into computational models of GO membranes to simulate interlayer separation and water diffusivity, providing insights for applications.[^19^, ^20^, ^21^] Theoretical studies delved into aspects such as the excess surface charge in hydrated GO and the dynamic evolution of functional groups, employing *ab initio* molecular dynamics (AIMD) for a comprehensive understanding of GO in water.^[22]^

Despite these advances, there remains an inherent limit to the length and time scales accessible to AIMD. Machine learning (ML) based interatomic potentials provide an emerging alternative approach that promises much faster simulations while retaining quantum‐mechanical accuracy.[^23^, ^24^, ^25^, ^26^] In the context of carbon materials, ML‐driven simulations have been used to describe defective^[27]^ and fully amorphous graphene,^[28]^ the growth of carbon thin films,^[29]^ and the formation of voids in low‐density porous forms.[^30^, ^31^, ^32^]

In the present work, we show how one can rapidly explore a wide range of functional groups and disorder in GO materials by combining two recent innovations in atomistic ML (Figure 1). First, we use on‐the‐fly‐accelerated AIMD[^12^, ^33^, ^34^] to efficiently sample configurations for an initial training dataset. Second, we show that this approach can be used to kick‐start a much wider‐ranging exploration using state‐of‐the‐art neural‐network potentials. For the first task, we use CASTEP+ML[^12^, ^35^] coupled to the Gaussian approximation potential (GAP) ML framework;[^15^, ^36^, ^37^] for the latter, we use an equivariant neural‐network architecture based on the message‐passing atomic cluster expansion (MACE),[^38^, ^39^, ^40^] which enables highly accurate predictions beyond the system‐size limits of AIMD. A key point of our study is that those two principally different methodologies can be synergistically combined. The predictions of the final ML model agree remarkably well with experimental observations, showing promise for future applications to the chemistry of carbon‐based materials.

**Figure 1:**
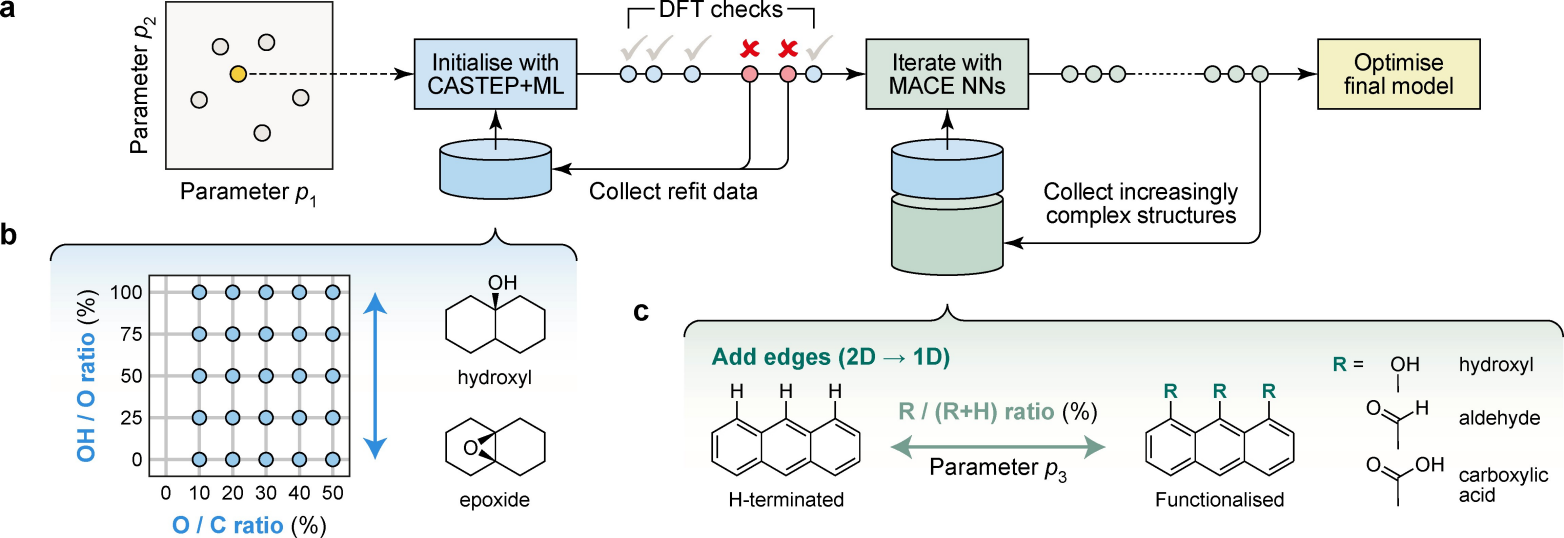
Accelerated exploration of functional groups in GO with machine‐learning‐driven simulations. (a) Schematic overview of the overall approach. We initialise the search for structures with CASTEP+ML trajectories, which combine first‐principles MD with on‐the‐fly fitting of ML potentials^[12]^ (blue). Once this initial generation is complete, iterative training kicks in, exploring increasingly complex structural spaces (green). The data are then used to train and optimise the final model (yellow). (b) Parameter space of 2D functionalised GO, with a schematic sketch of how the OH/O ratio controls the ratio of hydroxyl and epoxy groups in the initial structures. (c) Extension of the parameter space to include 1D structures (edges and ribbons), which can be hydrogen‐terminated or functionalised with different groups, R.

To sample the wide variety of possible GO structures, we define a space of *N* parameters, $𝒫=\left[p_{1},...,p_{N}\right]$ , that determines the composition of an initial candidate structure. Here, in line with existing knowledge in the field,[^1^, ^2^, ^3^, ^4^] we choose these parameters to be: (1) the ratio of O to C atoms in the initial sheet, determining the degree of oxidation; (2) the OH/O ratio, *i. e*., the concentration of hydroxyl groups relative to all O atoms; (3) the ratio of functionalised edges (−OH, −CHO, or −CO_2_H) to hydrogen‐terminated ones. We explored up to *p*_2_ in CASTEP+ML runs (that is, we functionalised only 2D graphene sheets), and up to *p*_2_ and then *p*_3_ in MACE iterations.

We started the process with 25 CASTEP+ML runs, at 300 K for 10 ps each, corresponding to the grid shown in Figure 1b. Most of those simulations (20 of 25) ran to completion; five terminated early due to erroneously lost atoms. The latter results are still valuable, as they contain high‐energy and ‐force structures which can be used to guide early models away from unphysical configurations during iterations. In all, 820 CASTEP+ML structures were used for the initial training dataset. Next, equivariant MACE potentials were trained in an iterative fashion that gradually extended the scope of the model: (i) exploring higher temperatures in MD runs, gradually increasing from 600 to 1,500 K; (ii) repeating the protocol at 1,500 K for the next four iterations; (iii) finally, exploring 1D structures at 1,500 K. After a total of 12 iterations, training structures with any force component >50 eV/Å were filtered out to further enhance the model. The final training dataset contains 3,016 simulation snapshots (605,204 atoms) and is described in detail in the Supporting Information.

We test the performance of our ML models on external data not seen in training: two AIMD trajectories of functionalised 2D sheets and 1D nanoribbons, respectively, as well as single‐point calculations for molecular (0D) fragments taken from Ref. [13]. Figure 2a shows how the force error—our main performance metric—evolves during iterations. As more data are added, the errors decrease for the 2D test set, as expected. For 1D and 0D structures, the errors are initially high since early models have not “seen” edges, specifically C−H bonds which are explicitly included only from model 10 onwards. Adding edge structures rapidly reduces the corresponding errors (dashed line in Figure 2a). The final force accuracy is similar across all benchmarks, just over 0.1 eV/Å.

**Figure 2:**
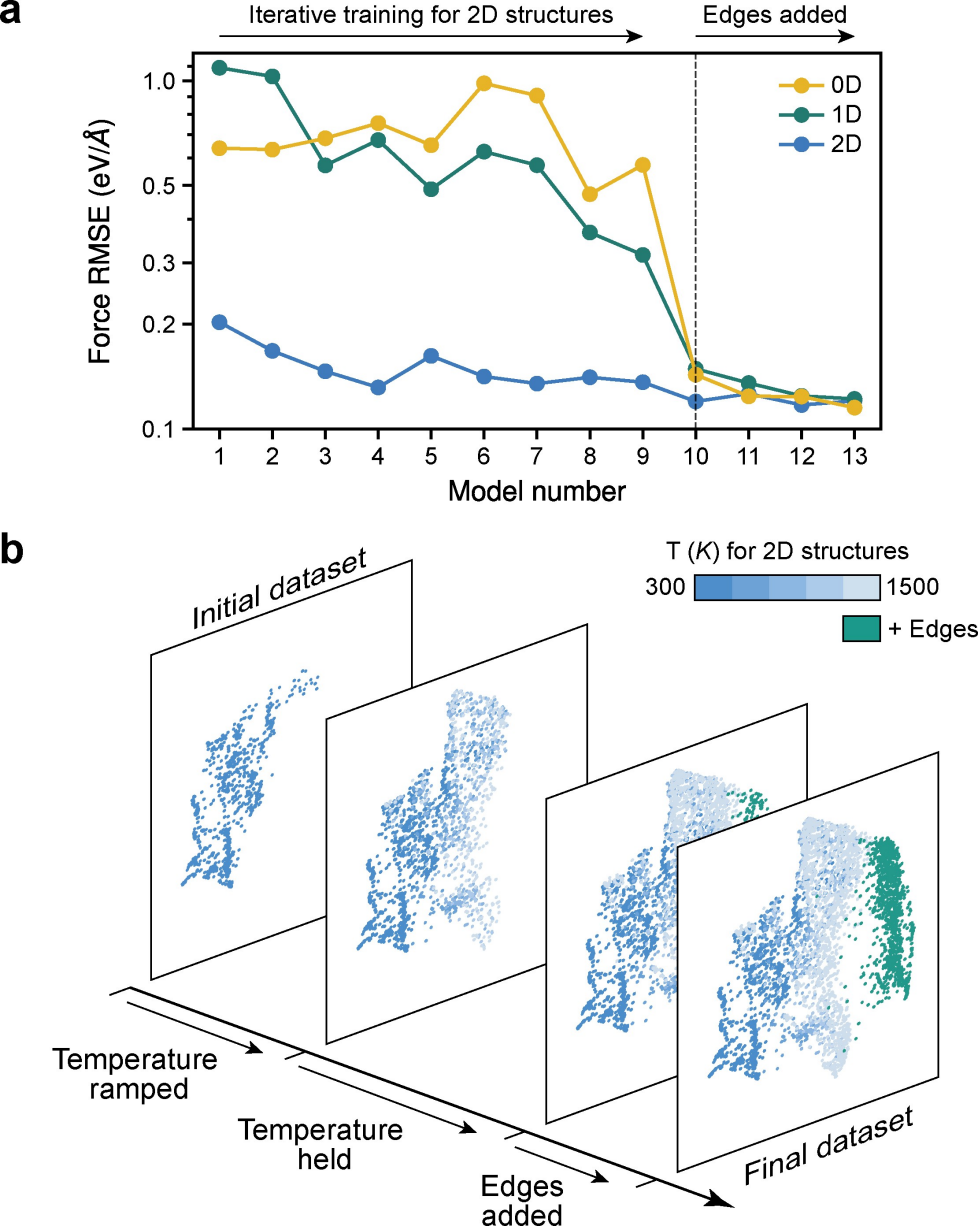
Training ML models for GO. (a) Root‐mean‐square error (RMSE) of forces predicted by iteratively trained MACE models. Errors are evaluated on an external set of DFT data not included in the training, comprising 100 snapshots each of a 2D sheet (blue) and a 1D nanoribbon (green) sampled in separate AIMD trajectories at 300 K, as well as 0D nanoflake structures (yellow) taken from Ref. [13] and re‐evaluated at the relevant level of DFT. (b) Structural diversity during iterative training. The different iterations are visualised by UMAP embedding^[14]^ of a kernel‐based structural similarity metric.^[15]^ Points are colour‐coded according to the temperature set in the MD simulation for 2D structures (blue), whereas they are shown in a single different colour for 1D edge structures (green).

To illustrate the gradual exploration of chemical and configurational space, we show four SOAP similarity maps[^15^, ^43^] in Figure 2b: the CASTEP+ML seed at 300 K, the dataset after gradually ramping to 1,500 K, the inclusion of the first edge structures, and the final dataset. The initial structures form two distinct clusters on the map; at higher *T*, one cluster grows and a third, smaller one appears. Finally, including edges adds a distinct set of structures (green).

We now describe an application of the final MACE model to a challenging problem in materials chemistry—namely, to large‐scale MD simulations of the thermal reduction of GO to rGO. This process involves a vast number of functional groups which transform and eventually disappear, accompanied by the evolution of gaseous species such as CO_2_. Experimentally, reduction temperatures of 1,100 °C yielded resistivity values of ~$10^{-5}\Omega$ m,^[44]^ on par with that of graphite.^[45]^ Understanding how functional groups evolve during thermal reduction could help to correlate the structure of the sheet with its properties. We show in the following that our ML‐accelerated approach can provide such an atomic‐scale understanding.

Our starting structure is a partially disordered, fully sp^2^‐bonded graphene sheet with 10,368 atoms (17.7×15.3 nm^2^ in a single layer). The sheet was generated using Monte‐Carlo bond switching driven by ML local‐environment energies, following Ref. [28], and then functionalised with $𝒫=\left[0.4,0.5,0\right]$ , raising the atom count to 16,645. This structural model represents features of GO including the topological disordering of the carbon backbone (presence of non‐6‐membered rings), although it is constrained to three‐fold coordination for all carbon atoms, and therefore does not initially contain large pores.

The thermal reduction was studied in three independent MD simulations at temperatures of 900, 1,200, and 1,500 K, respectively. The structures were rapidly heated over 100 ps and then held at the respective annealing temperature for 1.9 ns. We note that experimental protocols for thermal reduction of GO span a wide range of parameters: temperatures from 80 to 1,100 °C^[44]^ and times from 10 minutes^[46]^ to 5 days.^[17]^ Computationally, we are limited by the timescales accessible to MD (on the order of nanoseconds); thus, more aggressive heating is used to overcome local energy barriers.^[47]^ We found that annealing at 1,500 K yields a structure in good agreement with experiment, which we discuss below; results for the other MD runs are given in the Supporting Information.

Figure 3a shows the rGO structural model during annealing at 1,500 K. “Graphene‐like” regions, shown in dark grey, form small islands embedded within disordered and porous regions. (We quantify “graphene‐like” content through polyhedral template matching, a method to identify crystal‐like local environments.^[41]^) This result agrees qualitatively with electron microscopy images clearly showing amorphous regions together with holes and pores in the structure.^[48]^

**Figure 3:**
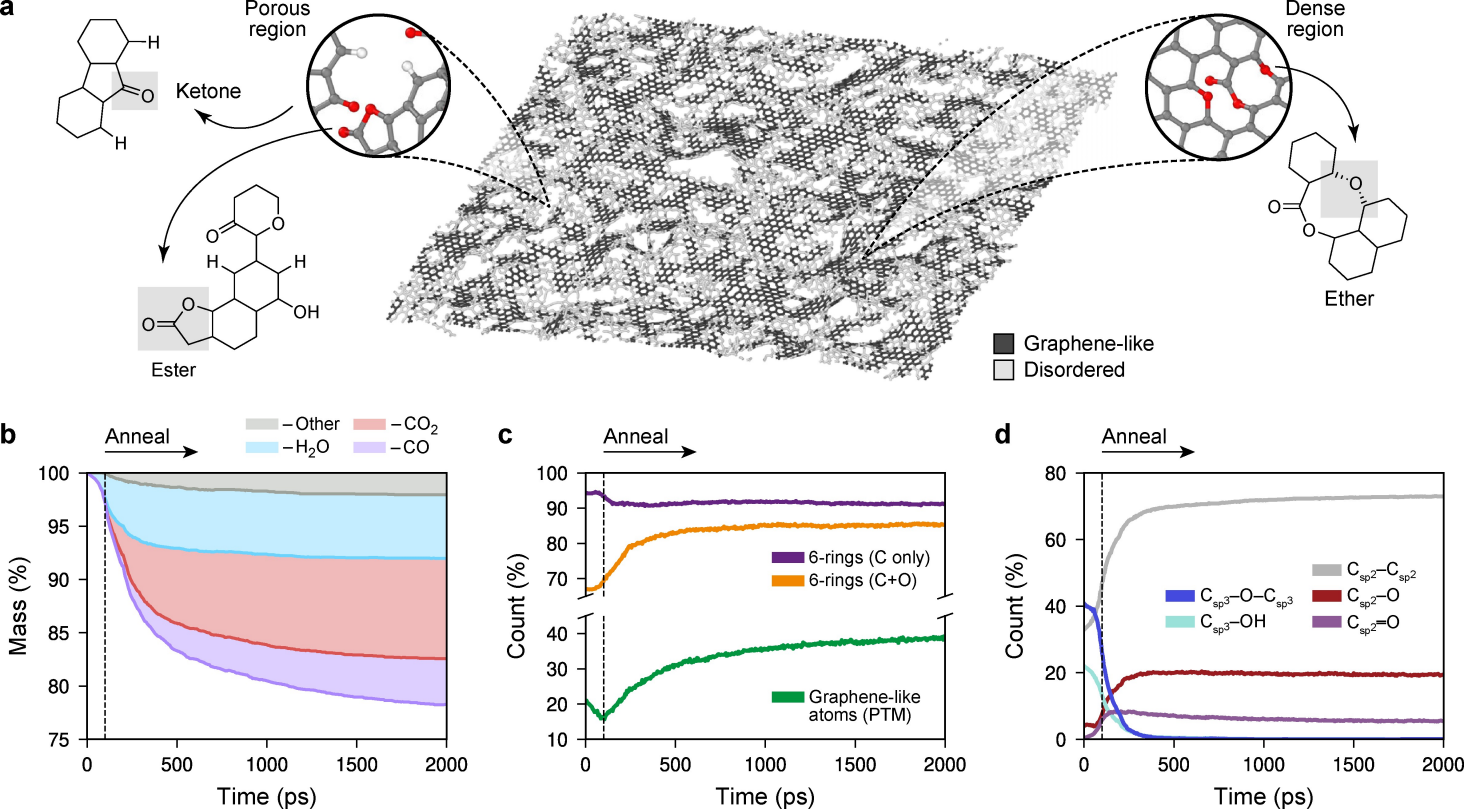
A large‐scale structural model of reduced graphene oxide (rGO) generated through simulated thermal reduction. (a) The rGO structure after 1.5 ns of simulation time (3 million timesteps). The dark grey regions highlight graphene‐like regions identified using polyhedral template matching (PTM).[^41^, ^42^] Insets show close‐ups with C atoms in grey, O in red, and H in white. (b) The change in mass of the GO sheet as it is thermally reduced in the 1,500 K simulation. The three most common leaving molecules (H_2_O, blue; CO_2_, red; CO, purple) are tracked in the stacked plot, along with other species (grey). (c) Evolution of structural indicators: the count of 6‐membered rings (shown separately for only C‐based rings, purple, and for all rings, orange), and the count of graphene‐like atoms identified by PTM. (d) Evolution of functional groups bonded to sp^2^ and sp^3^ carbon atoms, respectively, obtained using a topological bond‐counting algorithm.

The formation of this structure is accompanied by a mass loss of >20 % as gaseous species leave the surface (Figure 3b), which can be qualitatively correlated with thermogravimetric experiments.^[49]^ Initially, as the temperature ramps up over the first 100 ps, nearly all mass loss is due to H_2_O (light blue shading in the stacked plot of Figure 3b). Having reached 1,500 K (dashed line), the first CO_2_ molecules detach—and this species, indicated by red shading, quickly begins to dominate the mass loss as the sheet is reduced. CO was also evolved in notable amounts (magenta); other gaseous species such as OH, C_3_O_2_, H_2_O_2_, etc., were occasionally observed but were mostly rare and short‐lived.

In addition to the mass loss, our simulations allow us to address the changes in the structure of the GO sheet itself. Figure 3c shows the fraction of “graphene‐like” atoms in ordered local environments: initially, their percentage decreases, correlating with the loss of H_2_O; then, as CO_2_ is released, there is a clear and concomitant increase in graphenic content. These observations agree with experimental findings where CO_2_ and, to a lesser extent, CO loss leads to defects in the GO sheet,[^49^, ^50^, ^51^] inducing structural rearrangements and increasingly more graphene‐like environments. Interestingly, the count of 6‐membered rings containing only carbon decreases slightly (purple in Figure 3c), whereas if we consider all atoms in the sheet in the ring analysis, the 6‐membered rings increase from just above 65 % to ≈85 % (orange). This analysis suggests that oxygen atoms take part in substantial rearrangements during annealing, from sp^3^ environments perpendicular to the basal plane such as epoxide and alcohol groups, to sp^2^ environments parallel to the plane—for example, ethers and esters (cf. Figure 3a).

Beyond the overall “graphene‐like‐ness”, we can also trace the evolution of individual functional groups, enabling qualitative comparison with ^13^C NMR results from Ref. [49]. Figure 3d shows how the $C_{{\mathrm{sp}}^{2}}$ −$C_{{\mathrm{sp}}^{2}}$ count increases exponentially during the initial 100 ps—in contrast to the graphenic content, which decreases during this period (Figure 3c). This observation suggests that as water molecules leave the sheet, the carbon backbone will initially form defective rather than ordered sp^2^ environments. Then, during high‐*T* annealing, the sp^2^ count grows more gradually, indicating a transformation to a more graphene‐like carbon backbone, consistent with Figure 3c. The loss of oxygen‐based molecules (cf. Figure 3b) is clearly mirrored in a declining $C_{{\mathrm{sp}}^{3}}$ −O−$C_{{\mathrm{sp}}^{3}}$ (epoxide) and $C_{{\mathrm{sp}}^{3}}$ −OH (hydroxyl) count during the first 300 ps of the simulation. Concomitantly, $C_{{\mathrm{sp}}^{2}}$ −O groups form, which has been proposed as a mechanism by which the material drops in resistivity without a significant change in mass.^[49]^ Once the sp^3^‐bonded epoxide and hydroxyl groups are removed, the $C_{{\mathrm{sp}}^{2}}$ −O count remains at an almost steady state. Finally, the $C_{{\mathrm{sp}}^{2}}$ =O (carbonyl) count peaks at ≈250 ps before gently decreasing for the remainder of the annealing simulation.

With a quantum‐mechanically accurate description of the chemical structure in hand, the newly created structural models can now be analysed with advanced X‐ray spectroscopy predictions,[^52^, ^53^] as we have previously exemplified for small‐scale GO models (with DFT‐level predictions at the time).^[54]^ Here, applying the ML spectroscopy model of Ref. [52] reveals two peaks in the predicted X‐ray photoelectron spectroscopy (XPS) data for the initial structure (Figure 4a). The first peak corresponds to unmodified “sp^2^” carbon atoms, whilst the second relates to oxygen/hydrogen‐based functional groups, with the largest contribution arising from $C_{{\mathrm{sp}}^{3}}$ −O−$C_{{\mathrm{sp}}^{3}}$ (epoxide, dark blue) and $C_{{\mathrm{sp}}^{3}}$ −OH (alcohol, light blue) groups. The core electron binding energies (CEBEs) of all functional groups are shifted upwards from experimental reference energies due to the electronegativity of oxygen. The eventual removal of these oxygen‐based groups during annealing reduces the magnitude of the aforementioned CEBE shifts: all motifs decrease in CEBE.

**Figure 4:**
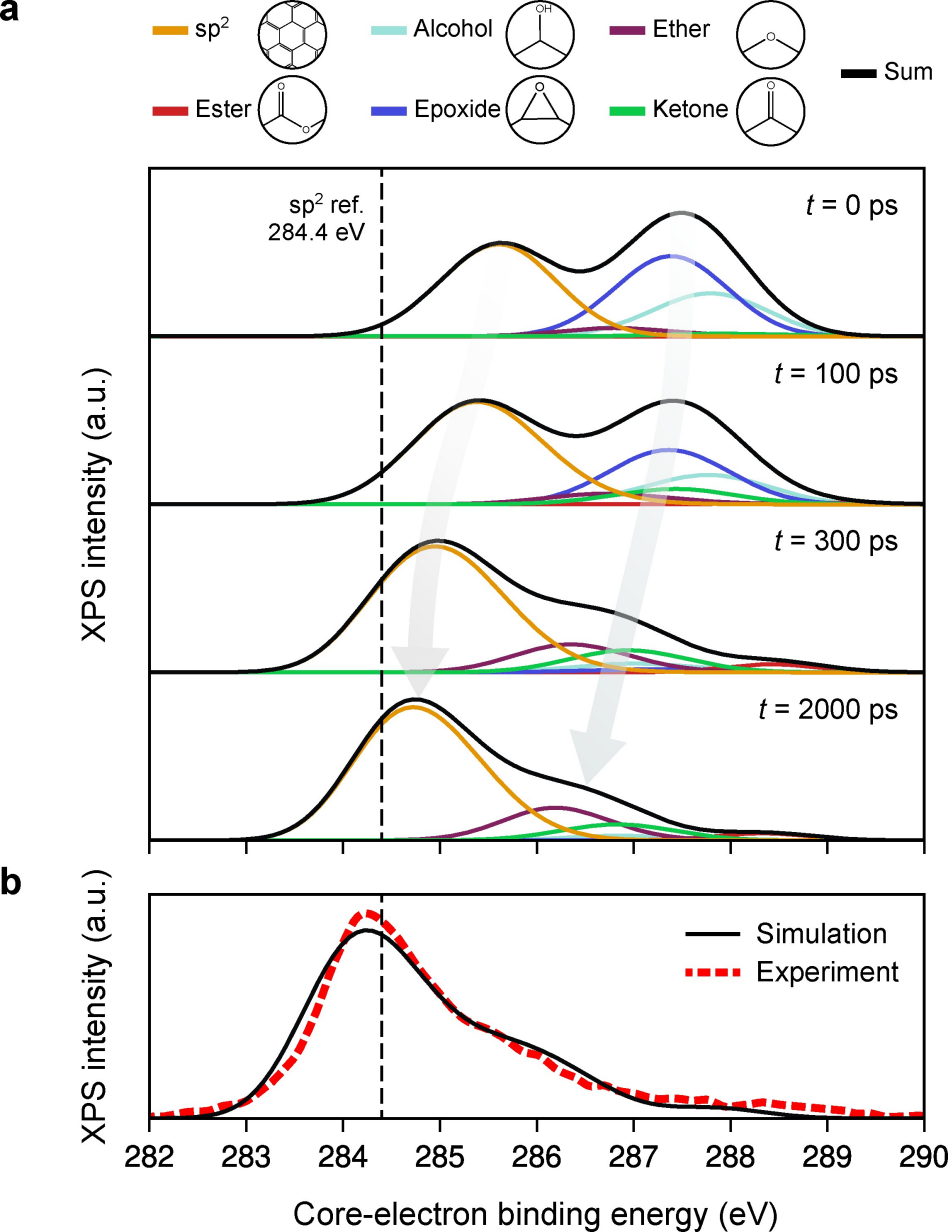
X‐ray photoelectron spectroscopy (XPS) predictions for the rGO structure annealed at 1,500 K. (a) ML‐predicted XPS spectra at different points of the simulation. The grey arrows indicate a clear shift to lower core‐electron binding energy values and also a reduction in the second peak during the simulation as oxygen‐based functional groups are removed. The vertical, dashed black line indicates the reference sp^2^ value at 284.4 eV. (b) XPS prediction for the final structural model obtained after full structural optimisation. The simulation data in panel (b) are shifted horizontally to align with the experimental data from Ref. [49].

Comparison with experimental data from Ref. [49], shown in Figure 4b, reveals good agreement between theory and experiment for rGO at 300 K (in air). Experimentally, XPS spectra use a fixed reference value for deconvolution, which does not take into account the effect of local interactions from electronegative species. As a result, the spectra are shifted accordingly to align well with experimental data. We refer the reader to Refs. [52] and [53] for a detailed discussion on this matter.

In conclusion, we have reported a computational approach to modelling and understanding the highly diverse chemical structure of GO, and we have shown an initial application to the thermal reduction of this material. Our work combines two recent developments in atomistic ML. For CASTEP+ML, we view its main advantage in this context to be in saving “human time”: it allows the researcher to create, from scratch, a chemically diverse training dataset to seed a new ML potential with minimal manual input.^[12]^ For MACE, our work builds on recent capability demonstrations,[^55^, ^56^] showing that this architecture can be combined with efficient dataset‐building workflows to readily deploy to new, challenging modelling problems in chemistry.

Looking forward, we expect this combined methodology to provide a powerful platform for further studies of GO materials. For example, the present proof‐of‐concept for XPS prediction during (simulated) structural transformations could motivate future *in situ* experiments—as an example of advanced, experimentally‐compatible modelling in which both the simulation *and* the XPS model are based on machine‐learned quantum‐mechanical data. Beyond the simulations of GO in vacuum reported here, the interaction of the material with water has been studied using empirical[^19^, ^20^] and DFT methods,^[22]^ and it would now be interesting to use ML‐accelerated modelling to more fully explore the nature of water between GO sheets—building on combined experimental and simulation studies in this area,^[57]^ and also on recent ML‐driven work on unconventional phases of water “sandwiched” between sheets of pristine graphene.^[58]^ A long‐term vision could be to use predictive ML‐driven simulations to find ways to optimise the nanoscale structure and thus the properties of GO materials—for example, porosity or catalytic activity—directly informing the preparation of samples in the laboratory.

## Conflict of Interests

The authors declare no conflict of interest.

## Supporting information

As a service to our authors and readers, this journal provides supporting information supplied by the authors. Such materials are peer reviewed and may be re‐organized for online delivery, but are not copy‐edited or typeset. Technical support issues arising from supporting information (other than missing files) should be addressed to the authors.

Supporting Information

## Data Availability

ata supporting this publication are available at https://doi.org/10.5281/zenodo.14066557.
