# Supplementary material for: Accelerated First‐Principles Exploration of Structure and Reactivity in Graphene Oxide
Source: Angew Chem Int Ed Engl. 2024 Nov 13;63(52):e202410088. doi: 10.1002/anie.202410088 (PMC11656143; doi:10.1002/anie.202410088)
Supplement: Supplementary file 1 — Supporting Information [file ANIE-63-e202410088-s001.pdf]

Angewandte Chemie
Eine Zeitschrift der Gesellschaft Deutscher Chemiker

Supporting Information

Accelerated First-Principles Exploration of Structure and Reactivity in Graphene Oxide

*Z. El-Machachi, D. Frantzov, A. Nijamudheen, T. Zarrouk, M. A. Caro, V. L. Deringer**

Supporting Information for

"Accelerated First-Principles Exploration of Structure and Reactivity in Graphene Oxide"

Zakariya El-Machachi[1], Damyan Frantzov[1], A. Nijamudheen[1], Tigany Zarrouk[2], Miguel A. Caro[2], and Volker L. Deringer*[1]

[1]*Inorganic Chemistry Laboratory, Department of Chemistry, University of Oxford, Oxford OX1 3QR, United Kingdom*
[2]*Department of Chemistry and Materials Science, Aalto University, 02150 Espoo, Finland*

*volker.deringer@chem.ox.ac.uk

Computational methods

ML acceleration for CASTEP. The CASTEP+ML scheme described in Ref. S1 was used to accelerate the sampling of the relevant configurational space (as compared to full *ab initio* molecular dynamics) and thus to construct an initial dataset for ML potential fitting. All DFT computations were performed at the Γ point and used the PBE functional with a plane-wave cutoff of 550 eV and a SCF halting criterion of $\Delta E < 10^{-5}$ eV at.$^{-1}$. A Gaussian smearing width of 0.1 eV was applied and all computations performed are non-spin-polarized.

For CASTEP+ML, the adaptive fitting method (increasing and decreasing the number of steps between DFT checks, n, depending on model performance; Ref. S1) was used, where $n_{\text{min}} = 1$ and $n_{\text{max}} = 10000$ with an adaptive scaling factor of 2. The tolerances used for refitting checks were an energy difference of < 0.01 eV at.$^{-1}$, a maximum force difference < 3.00 eV Å^{-1}, and a force RMSE of < 0.50 eV Å^{-1}.

The GAP models fitted by CASTEP+ML are based on a combination of 2-body, 3-body, and SOAP terms, similar to the C-GAP-17 model [S2], however with modified hyperparameters and much fewer sparse (representative) points particularly for the SOAP term. The GAP fitting string used for on-the-fly potential fitting is given in Listing 1 below.

Listing 1: GAP fitting string for CASTEP+ML runs.

```
default_sigma: "0.008 0.04 0 0"
descriptor_str: "distance_Nb order=2 cutoff=4.5 covariance_type=ard_se delta
    =2.0 theta_uniform=1.0 sparse_method=uniform add_species=T n_sparse=15 :
    distance_Nb order=3 cutoff=2.8 covariance_type=ard_se delta=0.5
    theta_uniform=1.0 add_species=T n_sparse=50 sparse_method=uniform : soap
    cutoff=4.5 covariance_type=dot_product zeta=4.0 delta=0.05 atom_sigma=0.5
     l_max=8 n_max=8 n_sparse=200 sparse_method=cur_points"
extra_gap_opts: "sparse_jitter = 1.0e-8"
```

MACE fitting. Equivariant MACE models [S3] were fitted on a single NVIDIA RTX A6000 graphics card in a Linux workstation. The final model required about 53 hours to train. We used the MACE code version 0.2.0, development branch, commit `55f7411`. The input used for MACE fitting is given in Listing 2 below.

Listing 2: MACE fitting input. "x" denotes the iteration number in the training and testing file.

```
CUDA_VISIBLE_DEVICES="$gpu_id" python mace/scripts/run_train.py \
--name="MACE_model" \
--train_file="structures/iter-x-train.xyz" \
--valid_fraction=0.10 \
--test_file="structures/iter-x-test.xyz" \
--config_type_weights='{"Default":1.0}' \
--model="ScaleShiftMACE" \
--hidden_irreps='128x0e+128x1o' \
--loss='huber' \
--r_max=3.7 \
--batch_size=30 \
--max_num_epochs=2000 \
--swa \
--default_dtype='float32' \
--energy_key='QM_energy' \
--forces_key='QM_forces' \
--stress_key=None \
--start_swa=1000 \
--swa_energy_weight=1000 \
--swa_forces_weight=100 \
--lr=0.001 \
--ema \
--ema_decay=0.99 \
--amsgrad \
--restart_latest \
--device=cuda \
--seed=123 \
```

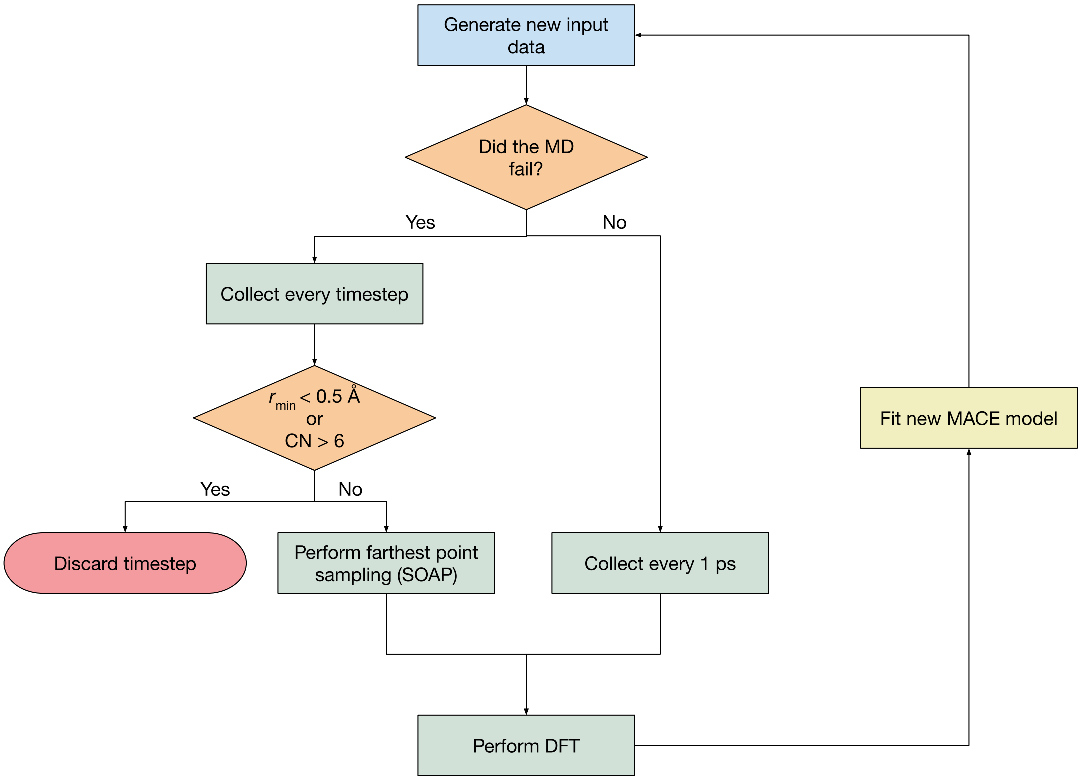

Figure S1: Flowchart outlining the process for selecting structures during iterative training.

Data filtering. The iterative process occasionally generated structures with very (even unreasonably) high energies and forces. We found that a process for filtering high-energy and -force structures throughout the iterative training process was important for the stability of early MACE models. Including highly unfavourable structures resulted in a less smooth regression—however, without inclusion of these unfavourable structures, some early models displayed instabilities and led to catastrophic failure during molecular dynamics (MD), such as lost atoms or nonphysical clustering. The approach to filtering structures during iterative training is illustrated in Figure S1.

Initially, we check whether a given MD run has completed without failing, in which case we simply collect structures at every 1 ps from the MD trajectory. However, if a run did fail, we investigate the trajectory in more detail. We heuristically define two criteria to determine whether a structure is fully nonphysical from the iterative MD (and should therefore not be included in the training dataset). We deem a structure to be fully nonphysical if it meets one of the following criteria:

- Any two atoms come closer than 0.5 Å (denoted "r_{min}" in Figure S1)
- The coordination number (CN) of any atom is greater than 6

The cutoff values were experimented with and proved to be sensitive to the types of structures filtered. Values of > 0.5 Å did not include enough unfavourable structures, whereas values of < 0.5 Å would include structures that then failed to converge in DFT.

Farthest point sampling (FPS) was used on the structures from the trajectory that remained after the filtering step. FPS was performed on the per-structure SOAP vectors constructed from the average atomic SOAP vectors of that structure. This enabled an automated and rigorous sampling of configurational space related to these failed MD runs. The final dataset was filtered of structures containing force components above 50 eV/Å.

Iterative training. MD simulations using the Atomic Simulation Environment (ASE) were used to generate configurations iteratively. The Nosé–Hoover thermostat in the NVT ensemble was used with an MD timestep of 0.2 fs. For the first four iterations, the temperature of the MD simulation was increased in increments of 300 K, starting from 600 K where model-1, which was trained on the CASTEP+ML data generated at 300 K, was used to drive MD across the parameter space and ending at 1,500 K for model-4, which had been trained iteratively from data generated from subsequent models. This iterative approach was chosen to enable a wider-ranging sampling of configuration space, without jeopardising stability in early models. We found that up to 1,200 K, the resulting MACE potentials were stable for at least 10 ps. At 1,200 K, the MACE potential at this iteration (model-3) was not stable for two runs at high O content and low OH/O ratio ($p_1 = 0.40$, $p_2 = 0.00$ and $p_1 = 0.50$, $p_2 = 0.25$), where model-3 incorrectly predicts a minimum in the potential energy surface, leading to unphysical clustering of atoms. This behaviour is somewhat expected during early stages of the iterative training of ML potentials, where the configurational space is still actively being explored. Furthermore, explicit models of isolated dimers are not included in the training data and thus extremely short-range interactions ($r \leq 0.5$ Å) are poorly described by early models. This was also observed at 1,500 K in the first instance (iteration 4) where two configurations failed ($p_1 = 0.50, p_2 = 0.00$ and $p_1 = 0.50, p_2 = 0.25$). 7 structures were removed from the final training set and 0 were removed from the test set.

Training and test sets. The total number of structures in the training set is 3,013 (+ 3 cells containing isolated atoms), for a total of 605,204 atoms. The testing set contains 800 structures (163,052 atoms). We also report the number of gaseous species from the training and testing set in Table S1. These gaseous molecules were identified using the average ionic radius to build

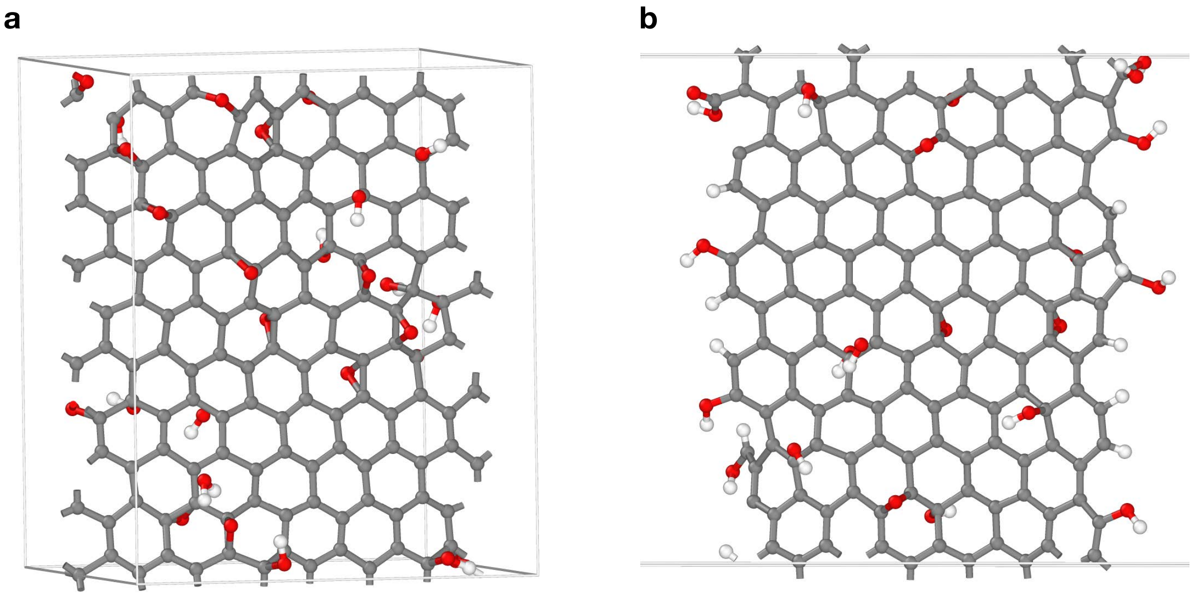

Figure S2: Representative structures from the training set. Panel (a) shows an example of a 2D structure whereas panel (b) shows an example of a 1D structure. Grey atoms are C, red atoms are O, and white atoms are H.

Table S1: Total gaseous species count from training and test set as determined through topological bond counting using pairwise cutoff radii and cluster analysis within OVITO [S4].

Gaseous Species	OH	H_2O	Other	CO	H	CO_2	O	O_2
Training Set Count	853	782	46	32	27	18	16	13
Test Set Count	213	213	13	9	8	7	4	2

pairwise cutoffs (with the exception of C–O, which required an increased cutoff of 1.85 Å, see Figure S13 below) to define bonds. These bonds were passed to the cluster analysis tool within OVITO [S4] to identify unique cluster fragments which could then be separately counted.

We note a relatively high count of OH and H_2O species in comparison to CO_2 and CO is observed due to the short MD time and high MD temperature (1,500 K for iter-3 and beyond) during iterative training. As seen in the main text in Figure 3, H_2O groups are lost early on during the simulation, whereas CO_2 dominates later on. Species labelled 'Other' are generated due to the high temperature and are short-lived.

Production MD. The same MD parameters as in the iterative training process were used for the production runs characterised in Figure 3, with the exception of the timestep, which was increased to 0.5 fs. Three production runs were conducted at 900 K, 1,200 K, and 1,500 K for the same initial structure.

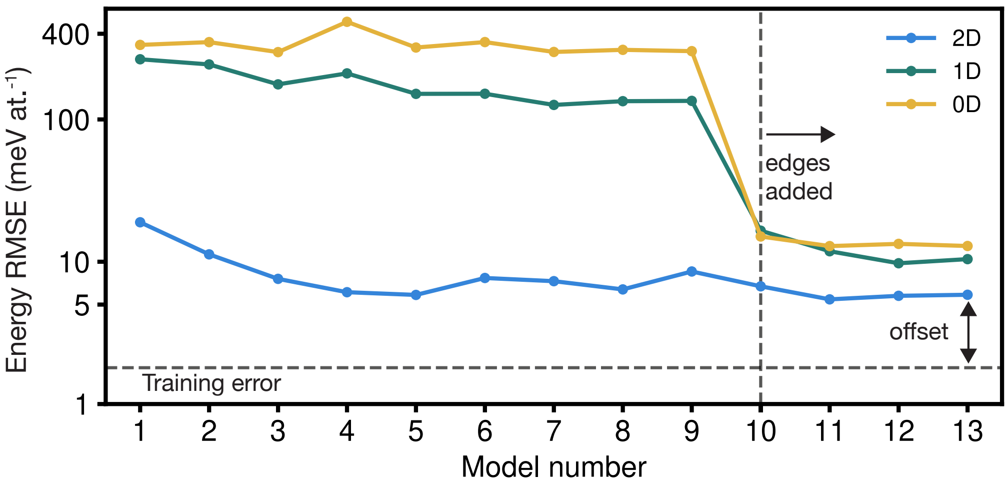

Figure S3: Energy RMSE for the external test sets discussed in the main text. The training error is shown as a dashed line at 1.8 meV at.$^{-1}$.

Energy RMSE. The energy RMSE values of the MACE models are characterised in Figures S3 to S6. Briefly, the energy predictions are offset by a notable amount, shifting the energy predictions up by approximately 6 meV at.$^{-1}$ for the external AIMD 2D test set and approximately 10 meV at.$^{-1}$ for the external AIMD 1D test set. The external 0D case (structures taken from Ref. S5) is not shown in the parity plots, as the structures have varying numbers of atoms, however the highest error is approximately 30 meV at.$^{-1}$ with the average being approximately 8 meV/atom as seen in Figure S6.

We believe that these offsets do not meaningfully impact the potentials' performance in dynamics since only the *relative* positions of minima and maxima are significant, not their absolute values. The variance of the data is perhaps another useful metric here, however this is not typically reported and thus we will refrain from doing so. However, it is important to note when reporting the energy RMSE, these potentials appear to perform worse than they actually do. We do not think that this offset is an issue specific to the MACE framework, and we will continue to investigate its origin.

At the end of the process, our final MACE model yielded an accuracy of 0.174 kJ mol^{-1} (1.8 meV at.$^{-1}$) for energies on the train set and 98.1 meV Å^{-1} for forces on the train set, the latter being in close agreement with the results for the external test set.

We also report the energy difference for the "internal" test set in Figure S6, which are defined as structures generated by the MACE model during iterative MD but not included in the training. In this internal test set, we have 800 structures and observe that there is no offset present as the

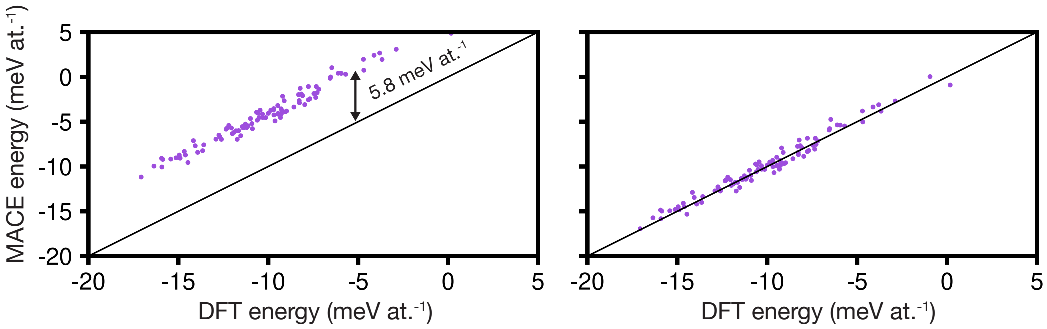

Figure S4: Energy parity plot for the 2D external test set. The left plot is the raw data, the right plot subtracts the shift to centre the plot.

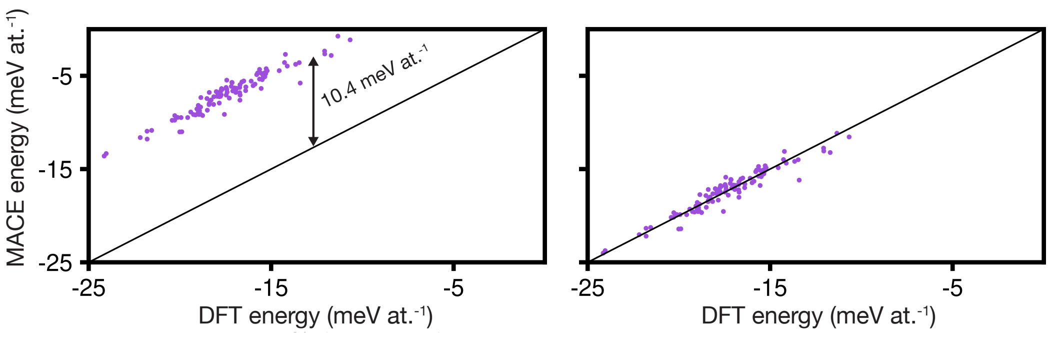

Figure S5: Energy parity plot for the 1D external test set. The left plot is the raw data, the right plot subtracts the shift to centre the plot.

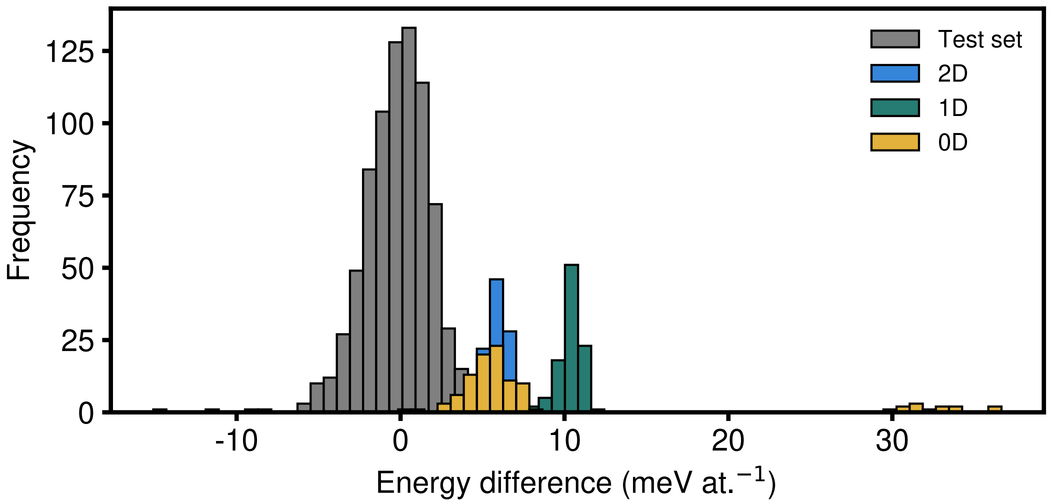

Figure S6: Histograms showing energy difference between final MACE model and DFT on the internal test set (grey); external AIMD 2D (blue) and 1D (green) test set and external 0D (yellow) test set using structures from Ref. S5.

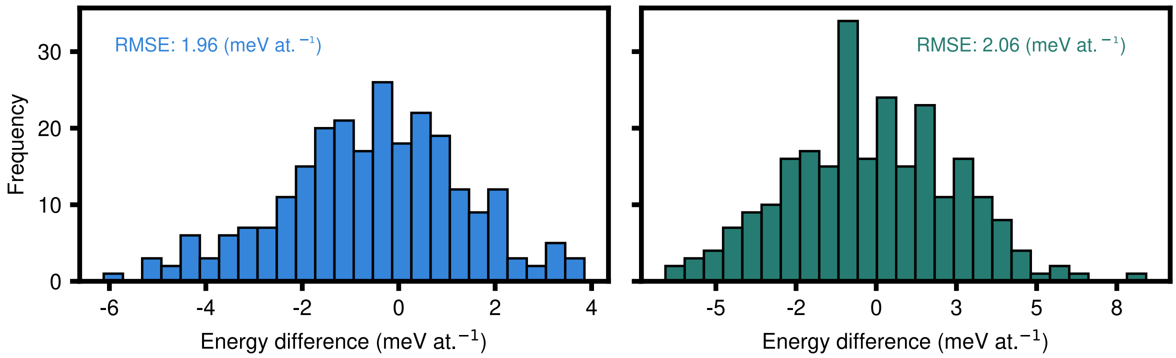

Figure S7: Distribution of energy differences with respect to DFT single-point computations, for separate test sets of 250 structures each, generated by MACE-driven MD at 1,500 K across $p_1 \in \{0.10, 0.20, 0.30, 0.40, 0.50\}$, $p_2 \in \{0.00, 0.25, 0.50, 0.75, 1.00\}$ (2D, blue), and $p_1 \in \{0.10, 0.20, 0.30, 0.40, 0.50\}$, $p_2 = 0.50$, $p_3 \in \{0.10, 0.20, 0.30, 0.40, 0.50\}$ (1D, green), respectively.

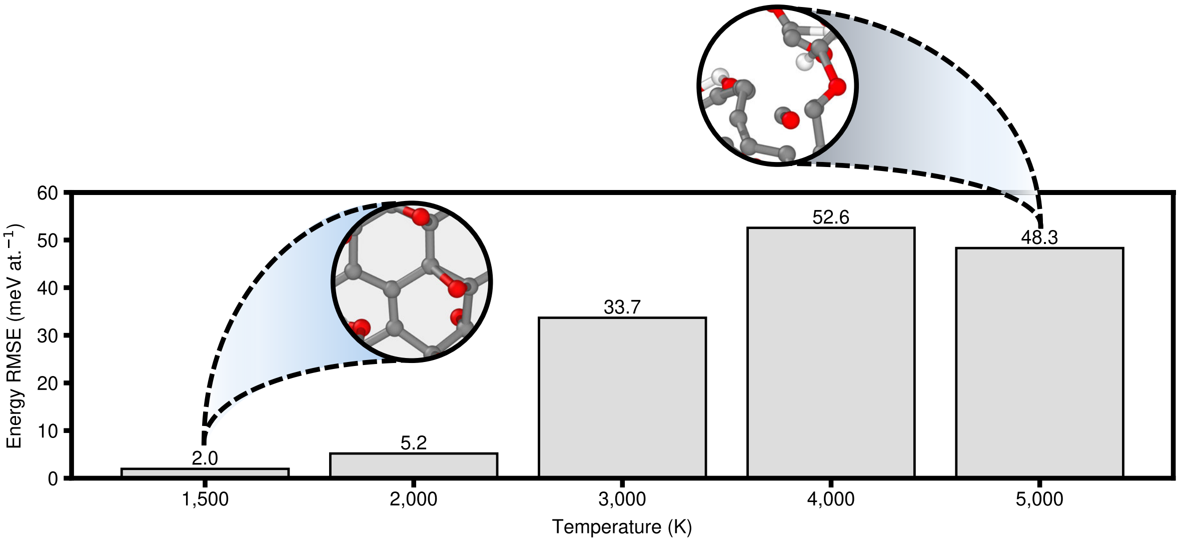

Figure S8: Energy RMSE relative to DFT single-point data for structures generated using MACE-driven MD in the high-temperature regime.

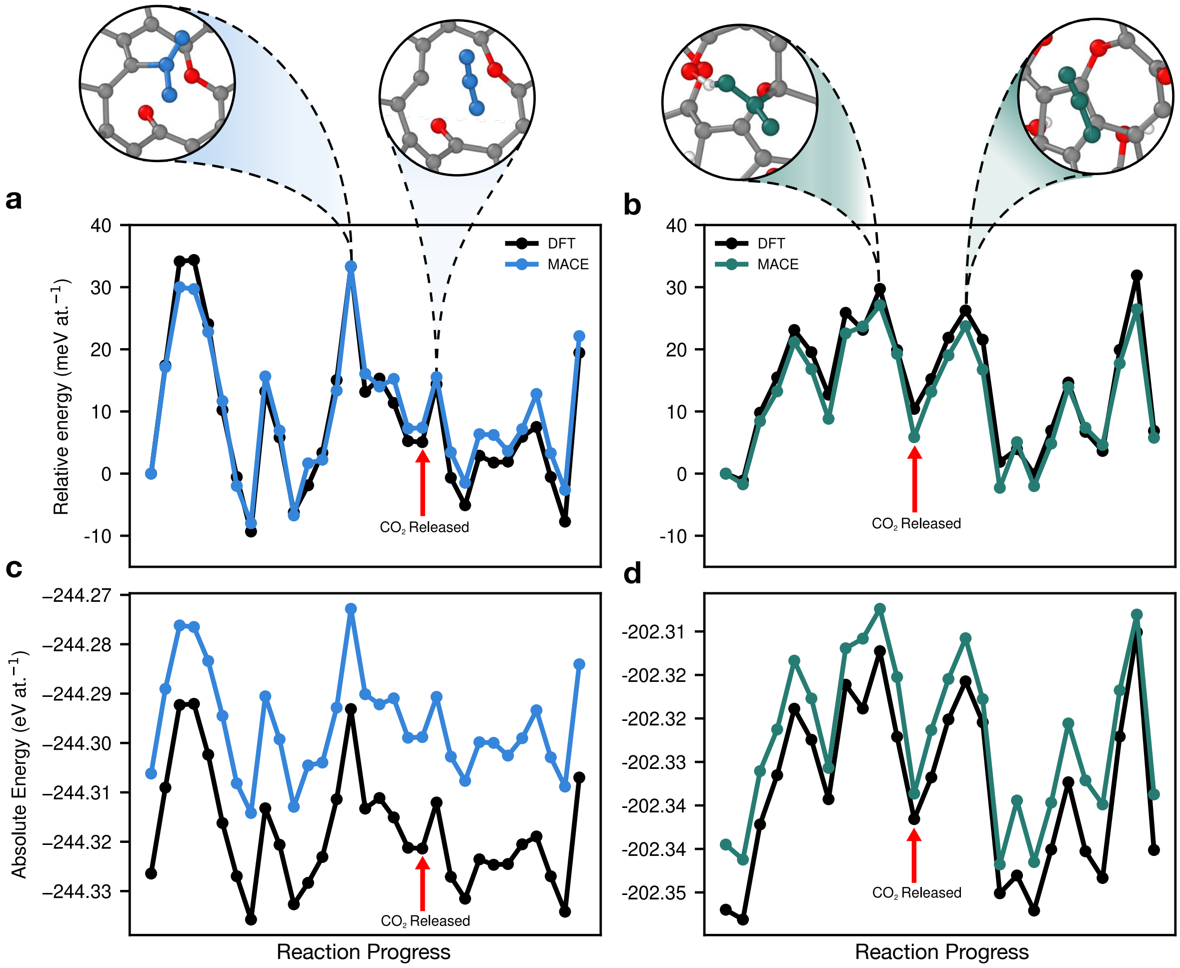

Figure S9: Energy profiles for two selected decarbonylation events from two separate 10-ps simulations. Panels (a–b) show the relative energy profiles of the decarbonylation events for a 2D (a, blue) structure and a 1D (b, green) structure compared with DFT single-point data (black), each shifted with respect to the first frame characterised here. Panels (c–d) show the absolute energy profiles.

data are centred around 0 meV at.$^{-1}$. As mentioned earlier, the 0D data show two regions, one centred at $\approx$ 8 meV at.$^{-1}$, which corresponds to H-terminated edges, and another region centred at $\approx$ 33 meV at.$^{-1}$, which corresponds to edges with no H atoms.

When MD is run using the MACE model and the results compared with DFT, we see that there is no offset present as seen in Figure S7. A set of structures spanning the p_1–p_2 (2D) and p_1–p_3 (1D) parameter space were used as an external test (data not explicitly included in training) and show the accuracy of the model on both 2D and 1D structures.

Additionally, we generate a set of high temperature configurations using the MACE model as seen in Figure S8. We ran MD for 10 ps using the same settings as the iterative training protocol and find that the model is stable in MD for 10 ps at these high temperatures. At 3,000 K and above, the sheet begins to disintegrate into chains and eventually gaseous species at higher temperatures. These configurations are very high in energy relative to the systems trained on and thus are outside of the scope of the training data. Whilst these energies are relatively high compared to 1,500 K and 2,000 K (also not trained on), we find that these errors are acceptable for these exotic configurations.

Finally, we track two decarbonylation events in a 2D and 1D structure as shown in Figure S9. The relative energy is in close agreement with DFT, as seen in Figure S9(a,b), yet the offset is apparent when using the absolute energies (c,d). This example is used to highlight that the relative energies are in good agreement with DFT. In order to thoroughly explore the reaction coordinate and barrier for a decarbonylation event, one would perform a nudged elastic band calculation which could constitute possible future work.

Polyhedral template matching. Polyhedral Template Matching (PTM) is used to identify atoms resembling simple crystalline structures at a local level [S6]. Within PTM, each input particle establishes a correlation between its local environment and the template of choice. If a correlation is identified, an RMSD (Root Mean Square Deviation) value is computed to measure spatial deviation from the ideal structural template, determining the match quality. In this work we used an RMSD cutoff value of 0.15 to determine whether a given local environment was "graphenic" or not. This method was used as implemented in OVITO 3.6.0 [S4].

Ring statistics. Ring statistics were determined using a shortest-path algorithm [S7] as implemented in `matscipy` [S8, S9].

Cell optimisation. The structures from the production runs were cooled down to 300 K over 100 ps and then underwent a full cell relaxation. This was done within ASE using the `FrechetCellFilter` class and the LBFGS optimizer. The force threshold was 10 meV at.$^{-1}$.

Methodology for X-ray photoelectron spectroscopy. The GW-corrected delta Kohn–Sham core-electron binding energy model from Ref. S10 was used to predict the C 1s core-electron binding energies (CEBE). This is a single `soap_turbo` [S11] model which is trained on neutral bulk delta Kohn–Sham CEBEs corrected by the difference of a GW core-electron binding energy and a charged delta Kohn–Sham core-electron binding energy on a configuration "carved" from an extended structure:

$$\Delta \mathrm{KS}^{0}_{\mathrm{ext}} + GW_{\mathrm{carv}} - \mathrm{KS}^{+}_{\mathrm{carv}}. \tag{S1}$$

Details of the methodology are given in Ref. S10.

Deconvolutions were performed by considering each structure at a particular timestep as an undirected graph, where edges are bonds, and nodes are atoms differentiated by species. Bonds cutoffs were defined in ASE using `natural_cutoffs(atoms, mult=1.2)`. A local subgraph was made for each carbon atom by adding edges and nodes initially from the atom's first neighbors, and if oxygen was found, adding the additional edges and nodes from the oxygen neighbors (which is necessary to discern epoxide and ether groups). Motifs were found by seeking subgraph isomorphisms between a reference dataset of motif graphs (e.g. aldehyde, alcohol, ketone etc). These were sought hierarchically, in the order: carboxylic acid, aldehyde, alcohol, carbonate, peroxide, ester, epoxide, ether, ketone, CH, CO_2, CO, sp^3, sp^2, sp.

Supplementary results

Mass-loss profiles (supplement to Figure 3b)

We carried out two further annealing simulations in parallel, starting from the same large-scale structural model as described in the main text, but now annealing at 900 and 1,200 K, respectively. The mass loss profiles for those lower-temperature simulations are shown in Figure S10. Overall, the change in mass at 1,200 K begins to plateau, converging at approximately 17%. This mass loss profile agrees qualitatively with experimental thermogravimetric data from Ref. [S12], where at the start of the thermal reduction, the mass loss follows a rapid exponential curve before reaching a linear phase for the remainder of the reduction process. In this way it is qualitatively similar to the 1,500 K simulation reported in the main text. The 900 K annealing run (left-hand side of Figure S10), however, shows much a much less pronounced mass loss which is largely due to loss of H_2O.

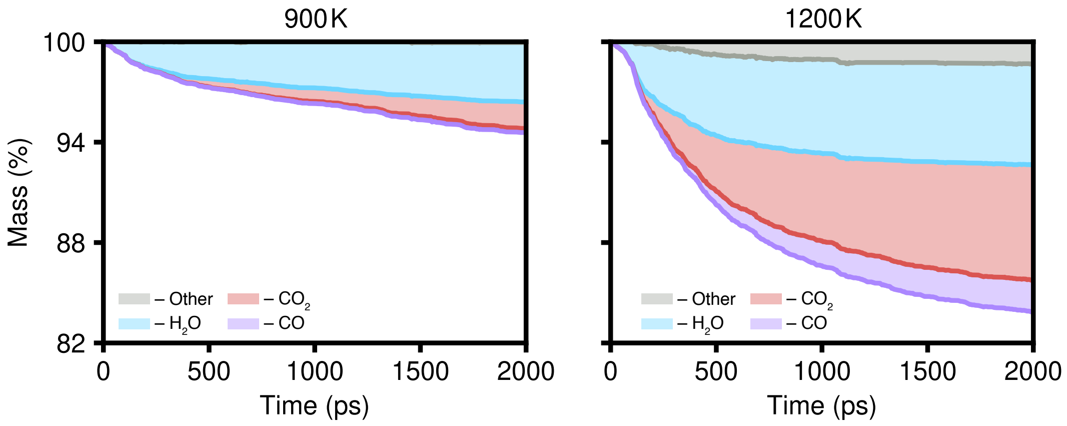

Figure S10: Mass loss profiles for the 900 K and 1,200 K simulations.

Graphene-like atoms and 6-membered rings (supplement to Figure 3c)

Figure S11 shows the evolution of the count of graphene-like motifs and 6-membered rings in the 900 and 1,200 K simulations. In our simulations, the "graphene-like" content eventually reaches ≈ 40 % for the 1,500 K MD run (Figure 3c) whereas it is still increasing for the 1,200 K run at just over 30 % (Figure S11). 900 K appears to be too low of a temperature to effectively reduce GO in nanosecond simulations: the degree of "graphene-like" similarity remains essentially steady, with only a very slight increase over 2 ns. The fraction of "graphene-like" atoms (dark grey) in combination with the percentage of 6-membered rings (blue) provides in-

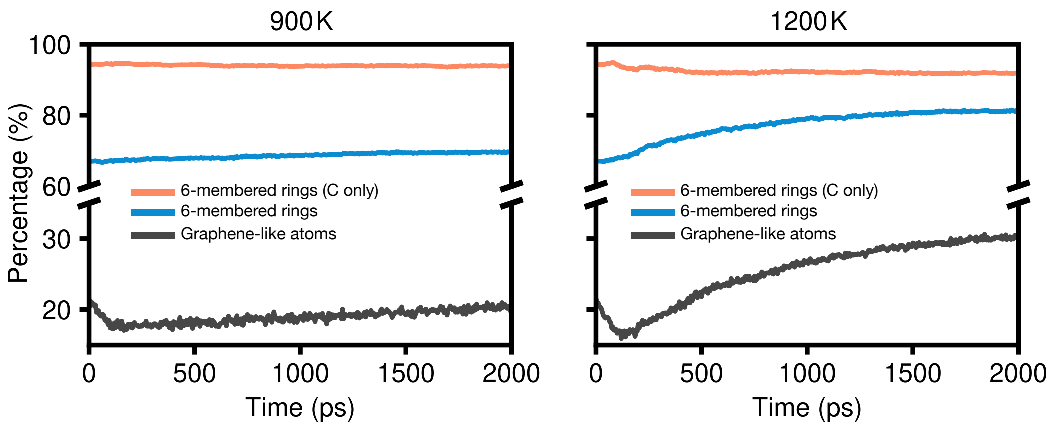

Figure S11: Graphene-like atoms and 6-membered rings profiles for the 900 K and 1,200 K simulations.

sight into the degree of crystallinity attained during the annealing process. We also show the percentage of 6-membered rings with only carbon atoms (orange) which is naturally quite high, but does not indicate how graphitic the structure is.

Annealing temperatures of 900 K appear to have no significant effect on the percentage of 6-membered rings which stays approximately constant. In addition to this, the count of "graphene-like" atoms decreases slightly during the initial heating phase. This is then followed by a very gentle linear increase which is still less than the initial percentage.

At 1,200 K, we observe more dynamic behaviour, where the percentage of 6-membered rings increases whereas that of 6-membered rings containing only carbon decreases, suggesting that more oxygen atoms are incorporated into the graphene network. This is supported by the X-ray photoelectron spectroscopy (XPS) data seen in Figure S14. In addition to this, the percentage of "graphene-like" atoms decreases at a faster rate when compared to the 900 K data over the heating period, before increasing at a faster rate before beginning to plateau after 2 ns. Overall, we see the percentage of "graphene-like" atoms increase with that of the 6-membered rings, as expected.

Functional groups (supplement to Figure 3d)

Tracking the functional groups during the annealing process provides insight into the mechanisms by which the structure becomes more graphene-like. For the 900 K simulation, there is a gradual decrease of sp^3 groups (dark and light blue) and a corresponding gradual increase of sp^2 groups (orange, red, purple). Overall, this profile further emphasises that 900 K is too low a temperature to effectively anneal GO structural models on the ns timescale using our

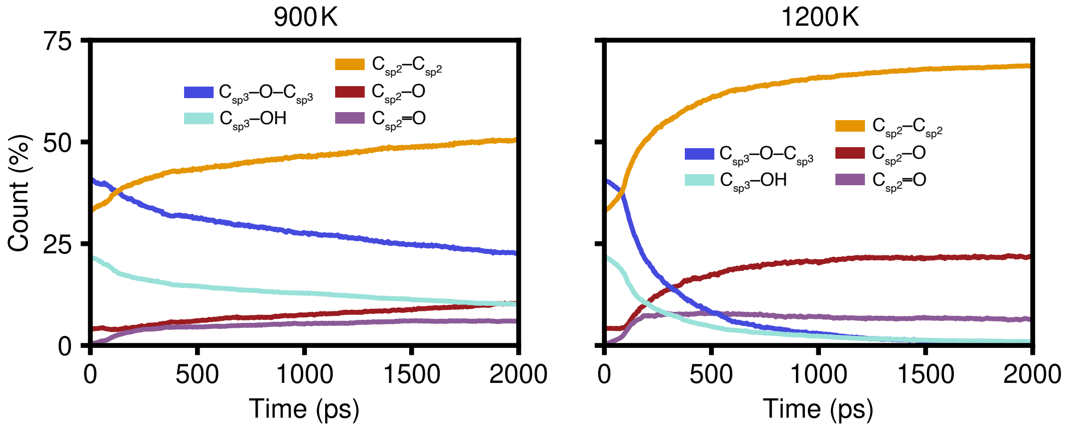

Figure S12: Functional group profiles for the 900 K and 1,200 K simulations.

model.

In contrast, the 1,200 K simulation follows a similar profile to the 1,500 K simulation, albeit at a slower rate. Here it is clear that sp^3 groups are nearly completely removed after 2 ns with a similar growth profile of sp^2 groups, including a characteristic steady-state period for C_{sp^2}–O groups.

We note that using the average ionic radius for the C–O bond ($C_{ionic}/2 + O_{ionic}/2$) gives oscillatory behaviour for the C_{sp^2}-C_{sp^2} count, as indicated by the black line in the top panel of Figure S13. As the defined bond cutoff is increased beyond the ionic radius (from 1.685 to 1.750 to 1.850 Å), we see the magnitude of oscillations decrease. We observe changes in the oscillatory behaviour during the course of the simulation, which may be due to gaseous species being removed and simulations restarted at some points. Much less pronounced oscillations are seen for the 1,500 K simulation, which is discussed in the main text (Figures S13 and 3d). For other species, all structural analysis was performed using the ionic-radius-based cutoff values.

X-ray photoelectron spectroscopy (supplement to Figure 4)

After the structures had been annealed, they were cooled down to 300 K over 100 ps and then underwent a full cell optimisation to relax the structure. XPS calculations were performed on these fully relaxed structures along with selected structures along the trajectory.

We summarize additional results in Figure S14. The top panel shows the shifted simulated spectra, where the shift has been introduced to better fit the experimental data (red, dashed). This is done since deconvolutions of experimental data are fixed at reference values (sp^2 =

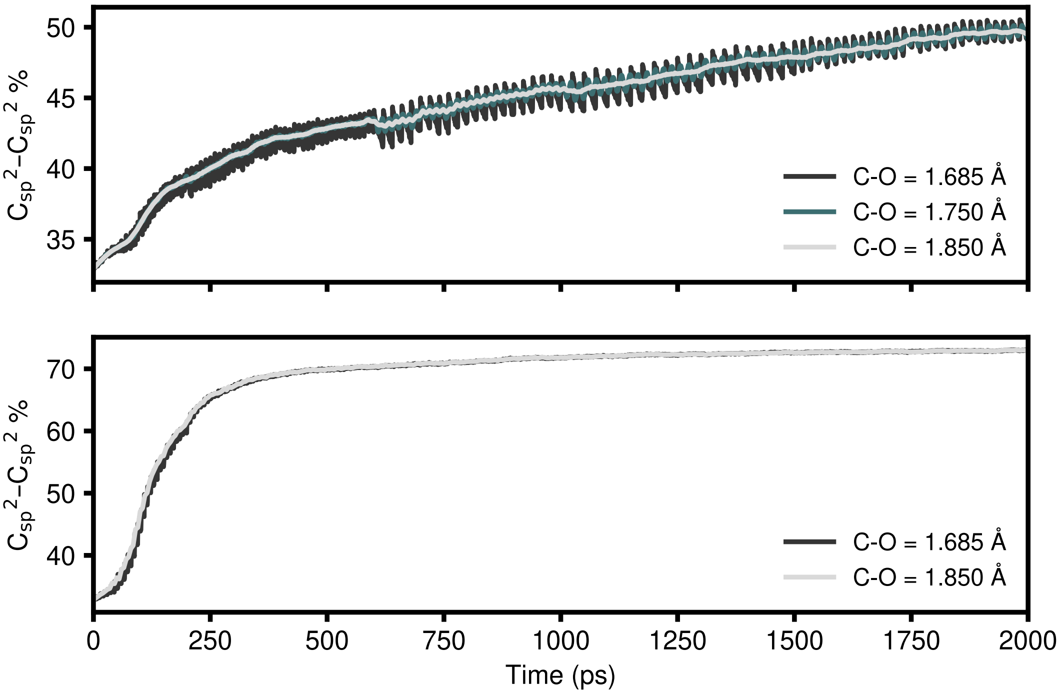

Figure S13: *Top:* Oscillations for the C_{sp^2}-C_{sp^2} count at 900 K, as evaluated using different C–O bond lengths for the cutoff definition. *Bottom:* Oscillations at 1,500 K. The data with a cutoff value of 1.850 Å are shown in Figure 3d of the main text.

284.4 eV, black dashed lines) to perform this task. In contrast, the simulated spectra are not constrained by this, and so we report both types of data in Figure S14; the data in the top panel are shifted towards the reference peak and the bottom panel shows the un-shifted simulation data. The experimental data taken from Ref. S12 have had their background removed and have been normalised in order to be compared to simulated spectra.

Both approaches provide useful information—the former giving comparison to experimental data, the latter providing information about the shifting of the peak towards the reference sp^2 value as oxygen is removed from the system after annealing at different temperatures. We have chosen to display only the six most important groups as other functional groups did not contribute substantially.

At 900 K, we see a spectrum which appears very different to the experimental data from Ref. S12 (red dashed line). This is due to the increased presence of oxygen groups in the structure, in particular, epoxide and alcohol groups which are yet to be removed. There are further contributions from ether, ester, and ketone groups. There is also a shift to higher CEBEs due to the increased electronegativity from the oxygen groups present.

At 1,200 K, the oxygen peak has now been reduced to a shoulder peak with the epoxide peak

being nearly removed entirely and the alcohol peak being greatly lowered. In contrast the ether group, ketone group, and ester group peaks have all increased greatly. This agrees with the wider trend of transitioning from sp^3 to sp^2. The shift down to the sp^2 reference peak is also observed in the bottom panel due to fewer oxygen groups being present and a greater presence of sp^2 environments.

Finally, at 1,500 K, we observe the best agreement with the experimental XPS data of Ref. S12. There are no significant contributions from epoxide environments and, in total, fewer oxygen groups present. This can be clearly seen by the reduction in the shoulder peak, giving closer agreement experiment than for the two other simulations at lower temperatures. Additionally, there is a closer shift to the reference sp^2 value, further highlighting the removal of oxygen. The results in Figure S14 therefore provide further justification that 1,500 K is an appropriate choice of annealing temperature for the simulations reported in the main text.

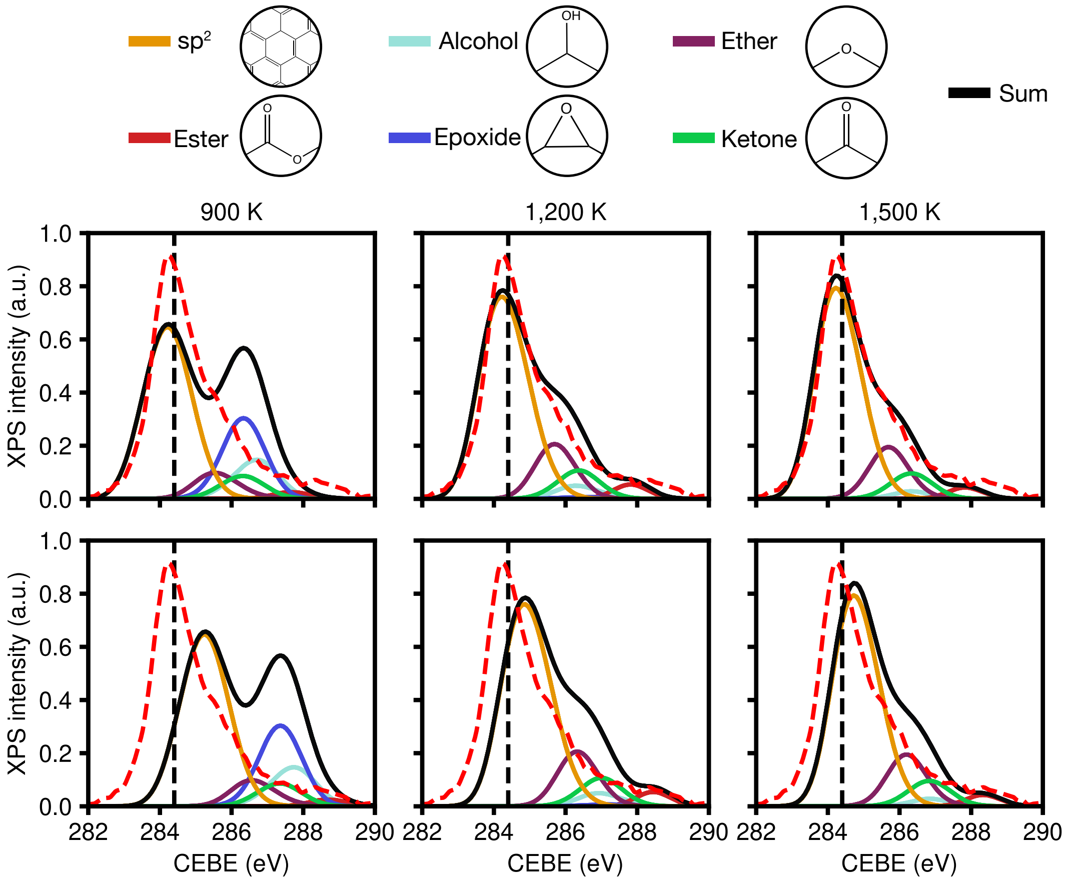

Figure S14: ML-model-predicted XPS spectra for the 900 K, 1,200 K, and 1,500 K simulations. **Top:** Shifted XPS spectra to experimental sp^2 reference value at 284.4 eV. The red dashed line shows experimental data taken from Ref. S12. **Bottom:** Unshifted XPS data showing the effect of electronegativity on CEBE values.

Supplementary references

[S1] T. K. Stenczel, Z. El-Machachi, G. Liepuoniute, J. D. Morrow, A. P. Bartók, M. I. J. Probert, G. Csányi, V. L. Deringer, *J. Chem. Phys.* **2023**, *159*, 044803.

[S2] V. L. Deringer, G. Csányi, *Phys. Rev. B* **2017**, *95*, 094203.

[S3] I. Batatia, D. P. Kovacs, G. Simm, C. Ortner, G. Csanyi, MACE: Higher Order Equivariant Message Passing Neural Networks for Fast and Accurate Force Fields, in S. Koyejo, S. Mohamed, A. Agarwal, D. Belgrave, K. Cho, A. Oh (Editors), *Advances in Neural Information Processing Systems*, volume 35, Curran Associates, Inc. **2022** pages 11423–11436.

[S4] A. Stukowski, *Model. Simul. Mater. Sci. Eng.* **2009**, *18*, 015012.

[S5] B. Motevalli, A. J. Parker, B. Sun, A. S. Barnard, *Nano Futures* **2019**, *3*, 045001.

[S6] P. M. Larsen, S. Schmidt, J. Schiøtz, *Model. Simul. Mater. Sci. Eng.* **2016**, *24*, 055007.

[S7] D. S. Franzblau, *Phys. Rev. B* **1991**, *44*, 4925.

[S8] J. R. Kermode, L. Pastewka, Matscipy (version 0.7.0), `https://github.com/libAtoms/matscipy` **2022**.

[S9] P. Grigorev, L. Frérot, F. Birks, A. Gola, J. Golebiowski, J. Grießer, J. L. Hörmann, A. Klemenz, G. Moras, W. G. Nöhring, J. A. Oldenstaedt, P. Patel, T. Reichenbach, T. Rocke, L. Shenoy, M. Walter, S. Wengert, L. Zhang, J. R. Kermode, L. Pastewka, *J. Open Source Softw.* **2024**, *9*, 5668.

[S10] D. Golze, M. Hirvensalo, P. Hernández-León, A. Aarva, J. Etula, T. Susi, P. Rinke, T. Laurila, M. A. Caro, *Chem. Mater.* **2022**, *34*, 6240.

[S11] M. A. Caro, *Phys. Rev. B* **2019**, *100*, 024112.

[S12] C. Valentini, V. Montes-García, P. A. Livio, T. Chudziak, J. Raya, A. Ciesielski, P. Samorì, *Nanoscale* **2023**, *15*, 5743.
